# Comparison of Opioid-Free Anesthesia Versus Opioid-Containing Anesthesia for Elective Laparoscopic Surgery (COFA: LAP): A Protocol Measuring Recovery Outcomes

**DOI:** 10.3390/mps3030058

**Published:** 2020-08-13

**Authors:** Anthony Eidan, Angela Ratsch, Elizabeth A. Burmeister, Geraldine Griffiths

**Affiliations:** 1 Anesthetic Department, Bundaberg Hospital, Wide Bay Hospital and Health Service, Bundaberg 4670, Australia; anthony.eidan@health.qld.gov.au; 2 School of Medicine, The University of Queensland, Brisbane 4072, Australia; 3 School of Nursing, Midwifery and Social Work, The University of Queensland, Brisbane 4072, Australia; liz.burmeister@health.qld.gov.au (E.A.B.); geraldine.griffiths@health.qld.gov.au (G.G.); 4 Nursing and Midwifery Services, Wide Bay Hospital and Health Service, Bundaberg 4670, Australia

**Keywords:** anesthesia-associated opioid, opioid-free anesthesia, contemporary anesthetic techniques

## Abstract

The administration of opioids is a central element in contemporary anesthetic techniques in Australia; however, opioids have a range of side effects. As an alternative, opioid-free anesthesia (OFA) is an emerging mode of anesthesia intended to avoid these side effects. This study is the first to publish the use of OFA in Australia and is conducted in a regional Queensland Health Service. The design will utilize a randomized clinical trial (RCT) to investigate the impact of OFA for patients having an elective laparoscopic cholecystectomy (*n* = 40) or tubal ligation (*n* = 40). Participant outcomes to be measured include: Quality of Recovery (QoR-15); Oral Morphine Equivalent Daily Dose (OMEDD) at 24-h post-operatively; time to first opioid (TTFO) dose; post-operative nausea and vomiting (PONV); Post Anesthetic Care Unit length of stay (PACU-LOS); and hospital length of stay (LOS). The findings may challenge the essentiality of opioids in the peri-operative period, which in turn would influence the future intra-operative management of surgical patients. Ultimately, a reduction in anesthesia-associated opioid use will support a more general decline in opioid use.

## 1. Introduction

Historical records indicate that humans have used opioids for at least 8000 thousand years as a mood enhancer, analgesic and hypnotic agent [1]. Evidence on Mesopotamian cuneiform tablets (c. 6000 BCE) refers to the medical properties of opium, and later, the Ebers papyrus (c. 1500 BCE) contains a record of the dried milky fluid from the poppy plant (opium) being used for headaches and as anesthesia [2]. The active ingredient in opium (morphine) was isolated in 1803, and its derivative, diacetaylmophine (heroin) in 1874. Opioids and their synthetic derivatives (Fentanyl) were first used in anesthesia in 1962 in Belgium and are now routinely administered via the oral, subcutaneous, intravenous, intramuscular, transdermal, epidural or via intrathecal routes specifically for their analgesic effects. Across the world, there has been a significant rise in the consumption of pharmaceutical opioids over the last twenty years. Australia now has one of the highest levels of use with almost 15 million opioid prescriptions dispensed in 2015 and with the use of high-potency opioids also increasing [3,4,5].

Traditionally in Australia, peri-operative analgesia has been provided by opioid analgesics, and current anesthetic practice is heavily dependent on opioid use during and after anesthesia for post-operative pain relief. However as White [6] indicates, ambulatory surgery involving high doses of opioids can be associated with increased post-operative complications including respiratory depression, paralytic ileus, nausea and vomiting, difficulty voiding, and pruritus. These can ironically increase length of stay, thereby reversing the objective of ambulatory surgery in providing rapid patient through-put. In addition, the intra-operative use of large bolus doses or continuous infusions of potent short-acting opioid analgesics (e.g., remifentanil) can increase post-operative pain due to their rapid elimination and the development of acute tolerance [7]. Accordingly, in the ambulatory peri-operative environment, anesthesiologists have explored the adjuvant use of non-opioid analgesics [8,9].

A non-opioid analgesic multimodal approach (opioid-free anesthesia (OFA)) is aimed at optimizing adjunctive options intra-operatively, utilizing anesthetic techniques targeting different neuroanatomical circuits and multiple neurophysiological mechanisms [10,11]. The pain (nociceptive) pathway commence with the A-delta and C-peripheral afferent nerve fibers. These fibers synapse on projection neurons in the dorsal horn of the spinal cord. The neurons then synapse in the brainstem including in the medulla at the nucleus of the tractus solitarius (NTS) [12,13]. The NTS initiates the autonomic response to nociceptive stimulus and then mediates, through the caudal ventral lateral medulla and the rostral ventral lateral medulla, (with projections to the adrenal medulla and the thoracolumbar sympathetic ganglia, the sympathetic output from the heart and peripheral blood vessels) [10,12,13]. In addition, the NTS synapses into the hypothalamus with the periventricular nucleus and supraoptic nucleus. The nucleus ambiguous synapses with the vagus nerve to the cardia sino-atrial node [12,13,14] to mediate the parasympathetic output. Blocking the autonomic nervous system response to nociceptive stimulus forms the major theoretical foundation of OFA [10,11,12,14].

Contemporaneous anesthetic practice calls for patient-centered approaches to assessing the recovery of patients after surgery. OFA has been shown to be an effective anesthetic technique for patients undergoing laparoscopic cholecystectomy [15] and, in addition, is associated with less peri-operative adverse events in cardiac surgery [16] and less nausea and vomiting in bariatric surgery [17] compared to opioid-containing anesthesia [18]. The measurement of Quality of Recovery (QoR) following anesthesia and surgery is broader than simply assessing pain management. It involves considerations of the patient’s post-operative status in comparison to their pre-operative status and includes return of self-care, household and work activities and mobility to the level pre-operatively. In measuring patient outcomes, there is a clear trend in favor of Patient Reported Outcome Measures (PROMs) i.e., the direct reporting of an individual’s health status by the individual [19]. In this clinical trial, the participant reported outcomes are central to the measurement of OFA therapeutic efficacy.

The research has been approved by the Royal Brisbane and Women’s Hospital Human Research Ethics Committee (HREC/2020/QRBW/62398), Queensland, Australia, and is registered with the Australian New Zealand Clinical Trials Registry (ANZCTR), trial number ACTRN12620000714987.

## 2. Experimental Design

### 2.1. Aim

This is an investigator-initiated, single site, prospective, randomized, parallel group, single-blind study, with concealed allocation of participants scheduled for elective laparoscopic tubal ligation or cholecystectomy surgery, randomized on a 1:1 basis to receive either a standard or opioid-free anesthesia protocol. The aim of this study is to compare clinical outcomes for participant’s receiving opioid-free anesthetic (OFA) with those receiving standard opioid-containing anesthesia.

### 2.2. Objective

The primary objective is to compare the quality of recovery from general anesthesia and surgery using the quality of recovery 15 item scale (QoR-15) [20]. The QoR-15 score (Table 1) is a shorter validated version of the QoR-40 and provides an efficient evaluation of post-operative recovery from the participant’s perspective with total QoR-15 score ranging from 0 (extremely poor recovery) to 150 (excellent recovery) [20]. Permission was obtained to use the scale in this research (P. Myles, personal communication, 11 February 2020). Minor amendments were made to two questions; the first to Question Seven changing the wording from “getting support”, to “able to get support”, and Question Eight changed from “able to return to work and usual home activities” to “able to do usual home activities”.

The secondary objectives and endpoints include comparing:

- Post-operative complications of medication administration including:
  ○ Respiratory depression;
  ○ Nausea and vomiting;
  ○ Delirium/hallucinations;
  ○ Pruritus;
  ○ Participant pain scores;
  ○ Functional activity scores;
  ○ Sedation level;
  ○ Local anesthetic toxicity.
- The use of analgesia:
  ○ Time post-operatively to first analgesia;
  ○ Type of analgesia;
  ○ Oral Morphine Equivalent Daily Dose (OMEDD).
- Time until post-operative mobilization.
- Length-of-stay in PACU.
- Hospital length-of-stay.
- Peri-operative adverse events.

## 3. Procedure

### 3.1. Inclusion, Exclusion, Recruitment and Consent

All patients presenting for an elective cholecystectomy or tubal ligation (with or without oophorectomy) will be identified by the research team as per Table 2 and will be recruited and consented according to Table 3. Participation will be until Day 1 post-operatively. No long-term follow-up will be conducted to assess chronic complications.

### 3.2. Sample Size, Randomization and Blinding

#### 3.2.1. Sample Size

Current literature reports that mean change QoR-15 scores (difference between baseline and Day 1 post-operative) is approximately 22 (on a scale of 0–150) with a standard deviation of four [20]. A difference in QoR-15 scores of eight has been reported as clinically significant [21]. To test (2-sided) the effect of a difference in mean change score of six with a standard deviation of four for OFA compared with non-OFA, with 80% power and alpha level of 0.01, the total sample size (30% compliance adjusted) required is 40 participants (20 required for both the OFA and the non-OFA groups). Allocations to treatment group will be stratified according to whether the procedure is a cholecystectomy or tubal ligation; therefore, the sample size will be doubled to enable combined and separate analyses of treatment groups between types of surgery. Accordingly, the total number of participants required will be 80, i.e., 40 in the OFA group (*n* = 20 OFA cholecystectomy and *n* = 20 OFA tubal ligation participants) and 40 in the non-OFA group (*n* = 20 non-OFA cholecystectomy and *n* = 20 non-OFA tubal ligation participants).

#### 3.2.2. Randomization

Eligible participants will be randomly selected to receive OFA surgery or non-OFA surgery on a 1:1 basis. Treatment allocations will be randomized into blocks of six and stratified by type of surgery (tubal ligation or cholecystectomy). Following consent, participants will be allocated a study participation identification number (ID). An envelope with corresponding ID will be opened by the anesthetist just prior to surgery. This envelope will contain the random allocation of OFA or standard treatment and the respective treatment protocol for the allocation.

#### 3.2.3. Blinding

Allocation to standard or OFA protocol will be completed prior to surgery as designated by the chief investigator. The participant and the outcome assessors (recovery and ward nurses) will be blinded to the allocation group. The chief investigator will not conduct any outcome assessments. The allocation/randomization arms will be as follows:

Group A: Investigational treatment—opioid-free anesthesia

Group B: Standard treatment—non-opioid-free anesthesia

### 3.3. Treatment Regimen

#### 3.3.1. Intra-Operative Participant Medication

Induction and maintenance anesthesia will be given according to Table 4 and Table 5. All participants who do not have contraindication to receive intravenous lidocaine will receive Lidocaine as per the guidelines in Table 6.

#### 3.3.2. Post-Operative Participant Assessment

The recovery room staff will be blinded to the participant’s anesthetic grouping. The intraoperative anesthetic charts (AARK) records will be printed without “anesthetic medications/agents administered intraoperatively”. The recovery nurses will receive the AARK print out with all other data, including procedure details, intraoperative monitoring and anesthetic notes. The recovery nurse will receive the Identification, Situation and Status, Background, Assessment and Actions, Recommendations and Responsibilities (ISBAR) handover from the anesthetic doctor/nurse. The recovery room staff will use the standard Post-anesthetic Pain Management Protocol (Supplementary Materials) for all participants. If there are any concerns during the recovery period, the recovery nurse will speak directly with the anesthetic registrar or consultant who delivered the intra-operative protocol for ongoing management for the participant.

### 3.4. Data Collection

#### 3.4.1. Participant Documentation

Table 7 details the data (and their definitions) to be collected for the following source documents:

- QoR-15 questionnaire (baseline and 24-h post-operatively).
- Preoperative anesthetic clinic assessment form.
- AARK.
- Post-anesthetic care unit (PACU) documents.
- National In-patient Medication Charts (NIMC).
- Queensland Hospital Admitted Patient Data Collection.
- Intravenous Patient Controlled Analgesia Order—Adult.
- Analgesia monitoring forms, intravenous, PCA, epidural and regional analgesia.
- Queensland Adult Deterioration Detection System (Q-ADDS).
- Clinical notes.

#### 3.4.2. Electronic Data Handling

Data will be collected and transcribed onto an electronic data collection sheet using Google Docs. A cloud-stored electronic database within Google Docs has been purpose built for this research by a research team member. Access is via two-factor authentication and password security. This methodology has been risk assessed by the Queensland Health Cyber Security team as meeting the needs for the storage of sensitive data.

### 3.5. Measures and Statistics

#### 3.5.1. Measures

Outcomes are measured at baseline, then at the induction of anesthesia and at Day 1 post-operatively. The post-operative assessment will be conducted on the ward or by phone (if participant is already discharged). The primary outcome is the 15-item quality of recovery (QoR-15) score (Table 1). Each question is measured on a Likert scale ranging from 0–10; where 0 = none of the time [poor] to 10 = all of the time [excellent]. Results are considered clinically significant if there is a change of score of eight [21].

#### 3.5.2. Statistical Methods

All participants randomized will be included in the analyses on an intention-to-treat basis. Ten percent of data collected and entered will be independently verified. If more than 0.5% disagreement between data collected/data entry exists, then a further 10% of data will be checked. This process will continue until no disagreement is found by the independent verifier.

Data distribution will be assessed. Descriptive statistics, including frequencies and percentages for categorical data, and means, standard deviations or medians, and ranges for continuous data, will be assessed to ensure randomization has succeeded, and to describe differences between groups.

Mean and total QoR-15 scores will be calculated per participant at baseline and on Day 1. QoR-15 change scores will be calculated to determine the difference between Day 1 and baseline scores for each participant. The effect of OFA versus non-OFA on recovery (QoR-15 score) will be evaluated using a mixed model with random effects (of individual participants) to estimate the effect of OFA on QoR-15 scores, accounting for baseline QoR-15 scores type of surgery and 24-h peri-operative opioid use. If there are no statistically significant random effects in the mixed-method model (tested using likelihood ratio test), univariate and multivariate linear regression models using the QoR-15 change score will be used for group comparisons and to estimate factors associated with outcomes. Interaction effects will be investigated and eliminated where possible through data transformation and the inclusion of interaction terms in the models. Stata 14 (StataCorp, College Station, TX, USA) and SPSS^®^ version 20.0 (IBM^®^ SPSS^®^ Statistics 20) for Windows will be used for all analyses. All analyses will be reported at the 0.05 (95% confidence interval) statistical significance level, unless Bonferroni corrected levels are appropriate.

## 4. Expected Results

The intent of this research is to consider the hypothesis that opioids are an essential component in the suite of anesthetic medications. In Western contemporary health care practices, the use of opioids during anesthetic is evidenced-based, or is it? The Australian and New Zealand College of Anesthetists is cognizant of the rising prescription and risk of opioids. Their position statement [22] around this matter strongly cautions against slow-release opioid prescription, particularly in the post-operative period. However, the millennia-long legacy of the “need” for opioids intra- and post-operatively appears unchallenged despite (or in spite of) the advanced science that exists around human anatomy and physiology, pharmacokinetics and pharmacodynamics. A growing body of evidence indicates that long-term opioid use begins with treatment of acute pain, including intra-operative and post-operative surgical pain [23,24,25]. Brat, et al. [26] show that among opioid-naïve patients, each refill and week of opioid use post-surgery is associated with a large increase in opioid misuse. This finding is confirmed by Macintyre, et al. [27] who suggest that post-discharge opioid use continues in some patients for some years after surgery.

The evidence provided by this research will contribute to the growing body of knowledge around the efficacy of newer opioid-free anesthetic techniques.

## Figures and Tables

**Table 1:** QoR–15 patient survey.

***How Have you Been Feeling in the Last 24 h?*** **(0 to 10, where 0 = none of the time [poor] and 10 = all of the time [excellent]**														
1.	Able to breathe easily	None of the time	0	1	2	3	4	5	6	7	8	9	10	All of the time
2.	Able to enjoy food	None of the time	0	1	2	3	4	5	6	7	8	9	10	All of the time
3.	Feeling rested	None of the time	0	1	2	3	4	5	6	7	8	9	10	All of the time
4.	Have had a good sleep	None of the time	0	1	2	3	4	5	6	7	8	9	10	All of the time
5.	Able to look after personal toilet and hygiene unaided	None of the time	0	1	2	3	4	5	6	7	8	9	10	All of the time
6.	Able to communicate with family or friends	None of the time	0	1	2	3	4	5	6	7	8	9	10	All of the time
7.	Able to get support from hospital doctors and nurses	None of the time	0	1	2	3	4	5	6	7	8	9	10	All of the time
8.	Able to do usual home activities	None of the time	0	1	2	3	4	5	6	7	8	9	10	All of the time
9.	Feeling comfortable and in control	None of the time	0	1	2	3	4	5	6	7	8	9	10	All of the time
10.	Having a feeling of general well-being	None of the time	0	1	2	3	4	5	6	7	8	9	10	All of the time
***How Have you Had Any of the Following in the Last 24 h?*** **(0 to 10, where 0 = none of the time [excellent] and 10 = all of the time [poor]**														
1.	Moderate pain	None of the time	0	1	2	3	4	5	6	7	8	9	10	All of the time
2.	Severe pain	None of the time	0	1	2	3	4	5	6	7	8	9	10	All of the time
3.	Nausea or vomiting	None of the time	0	1	2	3	4	5	6	7	8	9	10	All of the time
4.	Feeling worried or anxious	None of the time	0	1	2	3	4	5	6	7	8	9	10	All of the time
5.	Feeling sad or depressed	None of the time	0	1	2	3	4	5	6	7	8	9	10	All of the time

**Table 2:** Inclusion and exclusion criteria.

Inclusion	Exclusion
Aged 18–65 years	Pregnant women
Booked for elective laparoscopic cholecystectomy or tubal ligation (with or without oophorectomy	Body Mass Index > 35
Independent capacity to consent to participate in the trial	Allergy to opioids
American Society of Anesthesiologists (ASA) physical health score of I-II	Allergy to adjuvant drugs
	Persistent opioid use. Patients with OMEDD greater than 0 during previous week pre-op and taken for seven successive days or more including recreational drug use.
	Non-elective surgery
	Non-English speaking

**Table 3:** Recruitment and consent.

Step 1	The research nurse investigator will liaise with the theater booking team to identify potentially eligible patients scheduled on theater lists.
Step 2	At the patient’s pre-anesthetic appointment, the research nurse investigator will undertake an initial screening of those patient’s clinical records for inclusion and exclusion criteria.
Step 3	Potentially eligible patients will then be approached by the research nurse and the project outlined with them. Patients who initially indicate interest will be provided with an information sheet; the researcher nurse will talk about the project broadly and encourage the patient to ask questions. The research nurse will contact the principal investigator if response clarification is required.
Step 4	Those patients who have been provided with an information sheet will then see the anesthetist who will explain in detail the project, the mechanism by which OFA works, and the process of randomization.
Step 5	Patients will be assured that the treating doctors will follow all usual procedures to ensure the patient does not experience pain. That is, if it is evident that the patient is in pain, standard pain management protocols will be enacted during surgery and while the patient is in recovery.
Step 6	If the patient is comfortable with participation, the formal consent procedures will be undertaken. The random allocation as to whether the participant receives OFA or standard anesthesia will be explained to participants. It will also be explained that participants can choose to withdraw consent at any time and that the right of any participant to refuse to participate in the trial at any time without giving reasons will be respected and will not prejudice their further treatment.

**Table 4:** Induction of anesthesia.

Standard Anesthetic Induction	Opioid-Free Anesthesia Induction
Anxiolytic agent: 1 min prior to induction Midazolam 1–3 mg IV	Anxiolytic agent: 1 min prior to induction Midazolam 1–3 mg IV
Fentanyl 1–2 mcg/kg IV	Clonidine 1–3 mcg/kg IV in 3 divided doses (at induction, mid-surgery and end)
Lidocaine as per Table 5	Lidocaine as per Table 5
Propofol 1–2.5 mg/kg IV bolus or as per programmed Target Control Infusion (TCI)	Propofol 1–2.5 mg/kg IV bolus or as per programmed Target Control Infusion (TCI)
	Magnesium sulphate 40 mg/kg (ideal body weight IBW)
Dexamethasone 8 mg IV **and** Parecoxib 40 mg IV	Dexamethasone 8 mg IV **and** Parecoxib 40 mg IV
Neuromuscular blocker (NMB): Choice of any NMB is at the anesthetist’s discretion with NMB monitoring	Neuromuscular blocker (NMB): Choice of any NMB is at the anesthetist’s discretion with NMB monitoring
	Beta-blocker: Esmolol 10–50 mg IV bolus 20 s prior to intubation
On standby IV bolus medications: Ephedrine 3 mg/mL Phenylephrine 100 mcg/mL or Metaraminol 0.5 mg/mL	On standby IV bolus medications: Ephedrine 3 mg/mL Phenylephrine 100 mcg/mL or Metaraminol 0.5 mg/mL

**Table 5:** Maintenance of anesthesia: standard and opioid-free.

Maintenance of Standard Anesthesia	Maintenance of Opioid-Free Anesthesia
Lidocaine 1.5–2.5 mg/kg/h IVI as per Table 5	Lidocaine 1.5–2.5 mg/kg/h IVI as per Table 5
Opioids analgesics: Fentanyl 1–3 mcg/kg IV **or** Morphine 0.1–0.2 mg/kg IV	Magnesium sulphate: 20 mg/kg/h IVI (IBW)
Sevoflurane/desflurane 0.6–0.8 MAC (Minimal alveolar concentration) with BIS (Bispectral Index Score) target around 40 **or** Propofol TCI infusion (TCI 4-8 mcg/mL)	Sevoflurane/desflurane 0.6–0.8 MAC (Minimal alveolar concentration) with BIS (Bispectral Index Score) target around 40 **or** Propofol TCI infusion (TCI 4–8mcg/mL)
	Ketamine 0.25–0.5 mg/kg IV (bolus before end of surgery)
Paracetamol IV 1000 mg IV	Paracetamol IV 1000 mg
	Clonidine 1–3 mcg/kg IV in 3 divided doses (at induction, mid surgery and end)
Ondansetron 4 mg IV	Ondansetron 4 mg IV

**Table 6:** Intravenous lidocaine administration guidelines.

Criteria	Points Scored
Hypoalbuminaemia	1 point
Heart block (first degree)	1 point
Participant on Vaughan–Williams Type 1 medications; sodium channel blocker	1 point
Deranged liver function or hemihepatectomy	1 point
CKD stages 3–4	1 point
Age ≥ 65 years	2 points
Risk category	Sum of points scored
Low risk	<2 points
Intermediate risk	2–3 points
High risk	>3 points
*Low risk* Administer a loading dose of 1.5 mg/kg by intravenous injection over 2–5 min. Maximum loading dose of 150 mg. Followed by a maintenance intravenous infusion at 2.5 mg/kg/h until local anesthetic infiltration by surgeon after wound closure. *Intermediate risk* Administer a loading dose of 1.25 mg/kg by intravenous injection over 2–5 min. Maximum loading dose of 150 mg. Followed by a maintenance intravenous infusion at a rate of 2 mg/kg/h until local anesthetic infiltration by surgeon after wound closure. *High risk* Administer a loading dose of 1 mg/kg by intravenous injection over 2–5 min. Maximum loading dose of 100 mg. Followed by a maintenance intravenous infusion at a rate of 1.5 mg/kg/h until local anesthetic infiltration by surgeon after wound closure.	

**Table 7:** Definitions, variables and measures.

Measure	Definition	Assessment		
		Pre-op	Intra-op	Post-op
Administration of all peri-operative analgesia (theater/PACU and ward/unit) Time to analgesia Type of analgesia Route of analgesia Dose of analgesia	Time, type and dose of all analgesia peri-operatively		x	x
Adverse events (AE) and serious adverse event (SAE)	Adverse events are considered “serious” if they threaten life or function. Due to the significant information they provide, serious adverse events require expedited reporting. Serious adverse events (SAEs) are defined as any AE which: Results in death Is life-threatening Requires in-patient hospitalization or prolongation of existing hospitalization Results in persistent or significant disability/incapacity The term “life-threatening” in the definition of “serious” refers to an event in which the participant was immediately at risk of death at the time of event. It does not refer to an event, which hypothetically might have caused death if it were more severe. However, important clinical events may be considered a serious adverse experience if they require clinical intervention to prevent one of the listed definitions, e.g., an “allergic bronchospasm”, which required intensive treatment in an emergency room or at home. SAEs will be reported, whether or not it is considered related to trial treatment.		x	x
American Society of Anesthesiologists (ASA) score	The ASA score is a subjective assessment of a participant’s overall health that is based on five classes (I to V) and will be completed at eligibility screening. Only participants scoring I or II are eligible for the study. I Participant is a completely healthy and fit participant II Participant has mild systemic disease III Participant has severe systemic disease that is not incapacitating IV Participant has incapacitating disease that is a constant threat to life V A moribund participant who is not expected to live 24 h with or without surgery	x		
Analgesia monitoring form	Hospital analgesia monitoring form for intravenous, PCA, epidural and regional analgesia—adult	x	x	x
Clinical assessment	Standard clinical observations, temperature, pulse, blood pressure, physical examination and cognitive assessment, height and weight—BMI	x	x	x
Complications–anesthetic and surgical	Yes/no plus text description of complication		x	x
Date and time discharged from hospital	Participant notes, discharge summary			x
Date of surgery	Participant clinical notes		x	
Day 1 observations	Assessment conducted at 24 h (± two-hours) post-discharge from theater (i.e., admission to PACU time) on Day 1 post-operatively			x
Delirium/hallucinations	Any evidence/report of = Yes			x
Documentation confirming participant eligibility	Trial eligibility and consent form in participant notes	x	x	
Duration of surgery	AARK times.		x	x
Functional activity scores	Scored A = activity unlimited, B = activity mild to moderately limited by pain, C = activity severely limited by pain.	x		x
Local anesthetic toxicity	Any evidence of symptoms = Yes		x	x
Medical history/comorbidities	Participant clinical notes- text description	x		
Medication administered	Intra-operative, PACU and post-operative list of medications, dose, route		x	x
Oral morphine equivalent daily dose (OMEDD)	Oral morphine equivalent daily dose (OMEDD) allows for comparison of other opioids from all routes, oral, sublingual, transdermal, parental or rectal preparations to be summed and converted to an equivalent total oral morphine daily dose. Calculated with the Faculty of Pain Medicine (FPM), Australia and New Zealand College of Anesthetists (ANZCA) Opioid Calculator Participant self-report of prescription or recreational use of opioids pre-surgery. OMEDD scores will be calculated at baseline (for eligibility criteria) and at Day 1 to include surgery, PACU and ward/home	x	x	x
Overall length of stay (LOS)	LOS from theater start time to discharge from hospital (hours: minutes)	x	x	x
PACU	Post-anesthetic care unit			
PACU LOS	PACU LOS from automated anesthetic record keeping (AARK) admission time to PACU discharge time			x
Pain	Severity of pain is measured using the numerical rating scale, a points scale from zero to ten. Pain is documented as stated by the participant. 0 = no pain, 1 to 3 = mild pain, 4 to 6 = moderate pain and 7 to 10 = severe pain.	x		x
Pain score on Day 1	0 = no pain, 1 to 3 = mild pain, 4 to 6 = moderate pain and 7 to 10 = severe pain.			x
Pain score–highest pain score recorded while in PACU	0 = no pain, 1 to 3 = mild pain, 4 to 6 = moderate pain and 7 to 10 = severe pain.			x
Participant demographics and clinical characteristics	Collected at baseline including: Date of birth (age) Gender Smoking status Medical history/comorbiditiesCurrent weight and height (Body Surface Area/Body Mass Index)	x		
Quality of recovery form (QoR-15)	Evaluation of post-operative recovery from the participant’s perspective with total QoR-15 score ranging from 0 (extremely poor recovery) to 150 (excellent recovery). Assessment at baseline and at 24-h post-operatively.	x		x
Sedation levels	Sedation scored as 0 = awake, 1 = mild (easy to rouse), 2 = moderate (rousable but unable to keep eyes open more than 10 s) and 3 = severe (difficult or unable to rouse)			x
Time until post-operative mobilization	Time from admission to PACU until the time first mobilized			x
Time admitted to PACU	Arrival time in PACU			x
Type of surgery	Tubal ligation/cholecystectomy	x	x	
Urinary complications	Urinary system complications			x
Vital signs	All vital signs from baseline until hospital discharge including: Respiratory rate Blood pressure Oxygen saturation Pulse rate Temperature	x	x	x

## Supplementary Materials

The following is available online at https://www.mdpi.com/2409-9279/3/3/58/s1, Post-operative pain management of all patients.
