# Supplementary material for: Comparison of Opioid-Free Anesthesia Versus Opioid-Containing Anesthesia for Elective Laparoscopic Surgery (COFA: LAP): A Protocol Measuring Recovery Outcomes
Source: Methods Protoc. 2020 Aug 13;3(3):58. doi: 10.3390/mps3030058 (PMC7565777; doi:10.3390/mps3030058)
Supplement: Supplementary file 1 [file mps-03-00058-s001.pdf]

methods and protocols

MDPI

Comparison of recovery after surgery between opioid versus opioid-free anaesthesia for elective laparoscopic abdominal surgery

Post-operative pain management for all patients

Table S1: Post-Operative Pain Assessment in Post Anaesthetic Recovery Unit (PACU).

**PACU Patient Assessment**
Wait for patient to wake<br>Subjective and objective assessment<br>Assess comfort, ask:<br>"are you comfortable", "can you take a deep breath", "would you take pain relief for this at home"
Pain protocol if required as below

Table S2: Post-Operative Pain Management in Post Anaesthetic Recovery Unit (PACU).

**Standard Recovery Protocol**				
Contraindications: Sedation score ≥ 2<br>Respiratory rate < 8/min<br>Blood pressure < 80% of pre-operative reading<br>Allergy to specific opioid in question				
**Morphine Pain Protocol OR Fentanyl Pain Protocol**				
**Medication**	**Strength after dilution with 10 mls of 0.9% Sodium Chloride**	Dose	Frequency	Route
Morphine 10mg	1 mg per millilitre	As per protocol	As per protocol	IV
Fentanyl 100 micrograms	10 micrograms per millilitre			

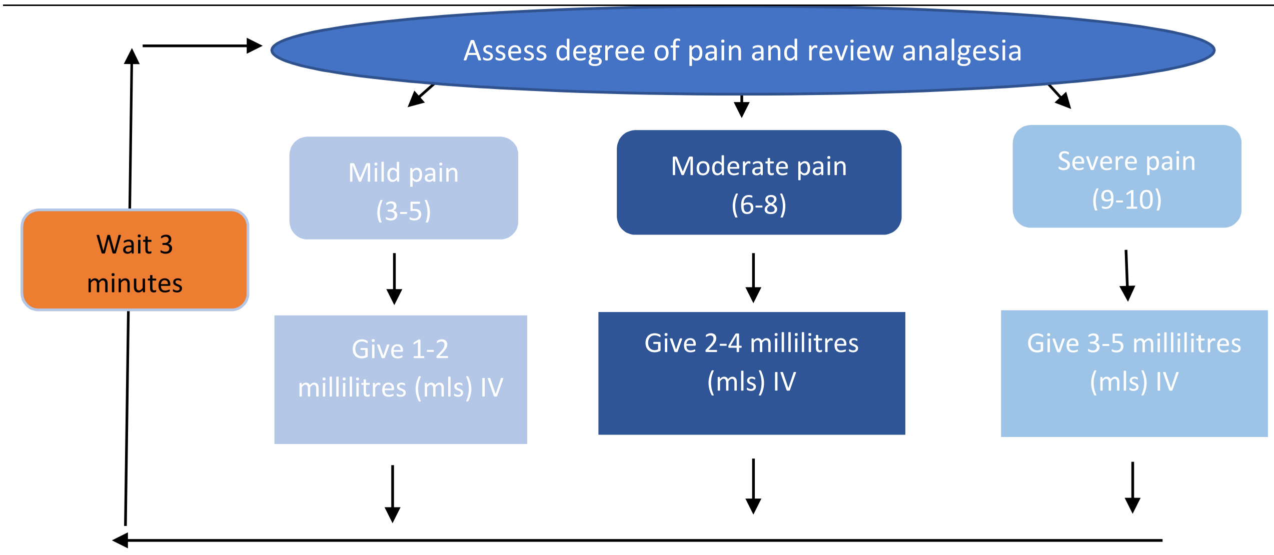

If no response after 10 millilitres (mls) of prescribed opioid, contact anaesthetist

Table S3: Pain Management on Discharge from Hospital.

Standard Discharge Medications
Paracetamol 1 gram QID for 5 days
Celebrex 200 mg BD for 5 days
Tramadol 50 mg IR (one to two tablets) every 6 hours as required for moderate to severe pain (10 tablets)<br>OR<br>Oxycodone 5 mg to 10 mg (one to two tablets) every 6 hours as required for moderate to severe pain (10 tablets)
